# BCL2 enhances survival of porcine pluripotent stem cells through promoting FGFR2

**DOI:** 10.1111/cpr.12932

**Published:** 2020-10-26

**Authors:** Zhenshuo Zhu, Qin Pan, Wenxu Zhao, Xiaolong Wu, Shuai Yu, Qiaoyan Shen, Juqing Zhang, Wei Yue, Sha Peng, Na Li, Shiqiang Zhang, Anmin Lei, Jinlian Hua

**Affiliations:** ^1^ College of Veterinary Medicine Shaanxi Centre of Stem Cells Engineering & Technology Northwest A&F University Yangling China

## Abstract

**Objectives:**

The establishment of porcine pluripotent stem cells (pPSCs) is still a critical topic. However, all pPSCs were failed to contribute to efficient chimeric pig and were extremely sensitive to changes of culture conditions. This study aimed to investigate the role of BCL2 in pPSCs and further explain the mechanism.

**Materials and Methods:**

Porcine BCL2 gene was cloned and overexpressed in porcine induce pluripotent stem cells (piPSCs). Digital RNA‐seq was performed to explain the mechanism of anti‐apoptosis. Finally, the cells carrying BCL2 were injected into mouse early embryo to evaluate its chimeric ability.

**Results:**

Here, we found that overexpression of porcine BCL2 gene significantly improved the survivability of piPSCs and the efficiency of embryonic chimerism, and did not wreck the pluripotency of piPSCs. Furthermore, the Digital RNA‐seq analysis revealed that BCL2, as a downstream gene of the PI3K signal pathway, enhanced the expression of PI3K signal pathway receptors, such as FGFR2, and further promoted oxidoreductases activity and lipid metabolism, thus maintaining the survival and pluripotency of piPSCs.

**Conclusion:**

Our data not only suggested that porcine BCL2 gene could enhance the survivability and chimeric ability of pPSCs, but also explained the positive feedback mechanism in this process, providing strong support for the chimeric experiment of pPSCs.

## Graphical abstract

Overexpression of porcine BCL2 gene promotes the expression of FGFR2 to resist cell death under conditions of stress. Furthermore, the survival rate and chimerism efficiency of porcine induce pluripotent stem cells were significantly improved.

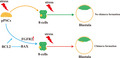

## 1 INTRODUCTION

Pig is an important domestic animal, which not only provides a large amount of meat for human, but also can be used as an ideal medical model due to its close similarity to humans in organ anatomy, physiology and metabolism.^1^, ^2^ Pluripotent stem cells (PSCs) can maintain self‐renewal and have the ability to differentiate into all cell types of biological individuals.^3^ Therefore, the derivation of porcine PSCs has future utility in biomedical and porcine breeding. However, porcine PSCs, including embryonic stem cells (ESCs) and induced pluripotent stem cells (iPSCs), still have great defects that lead it unavailable.^4^

At present, each of the porcine embryonic stem cell‐like cells (pESLCs) exhibited some features of pluripotency; however, these cells cannot maintain self‐renewal for a long time.^5^, ^6^, ^7^ Further investigation showed that only individual cell lines exhibited inefficient chimeras.^8^ Porcine‐induced pluripotent stem cells (piPSCs) can be obtained by ectopic expression of defined pluripotent factors and exhibited higher pluripotency compared with pESLCs.^9^, ^10^, ^11^ Unfortunately, most of piPSCs also failed to contribute to chimeras with some exceptions that depended on genomic polymerase chain reaction (PCR) analysis.^12^, ^13^ Therefore, the biggest drawback to the present pPSCs is that it is still hard to contribute to efficient chimeras and was extremely sensitive to changes culture conditions, although they can be passaged for a long time and differentiate into three germ layers after injection into severe combined immunodeficiency (SCID) mice.^4^ Culture conditions of pPSCs are more demanding, requiring growth factors and feeder, but even so it is prone to cell death and differentiation.^14^ This may be one of the main reasons why the chimeric experiments of pPSCs continue to fail. This is a major problem that must be faced if the field is to move forward.

Previous studies have shown that apoptosis is an initial barrier in the chimeric experiment. Inhibiting apoptosis can improve the survival ability to cope with stress conditions and the efficiency of embryo chimera formation, without affecting pluripotency of PSCs.^15^, ^16^, ^17^ One strategy to enhance the survivability of stem cells is recombinant overexpression of anti‐apoptotic genes or the addition of apoptotic inhibitors.^15^, ^17^ For example, Y‐27632, a selective Rho‐associated kinase (ROCK) inhibitor, has been shown to significantly diminish dissociation‐induced apoptosis of stem cells.^18^ The optimization of culture media for growth and survival can contribute to chimera formation in embryos.^19^

B‐cell lymphoma 2 gene (BCL2), one of the most particular interest is an anti‐apoptotic gene, was first identified in human follicular lymphoma.^20^ Many of the signals of improving survival converge at the BCL2 pathway. Previous studies showed that the expression of this gene can prevent apoptosis and reduces oxidative stress to improve the survival of human and mouse ESCs.^15^, ^17^ Further studies have shown that overexpression of BCL2 enables injected EpiSCs (epiblast stem cells) to survive and form chimeras.^16^ Furthermore, the overexpression of BCL2 enabled rat EpiSCs to contribute to mouse embryonic chimeras, thereby forming interspecies chimeras that could grow into adulthood.^16^ Therefore, the anti‐apoptotic ability of BCL2 significantly improves the formation of chimera, which is likely to have a similar effect in the chimerism test of porcine embryos.

We generated a porcine iPSCs that constitutively express porcine BCL2 and investigated the role of the BCL2 in piPSCs and interspecific chimeric embryos. We found that overexpression of BCL2 significantly decreases environmental stress‐induced apoptosis, resulting in enhanced embryoid body formation and enabling the piPSCs to contribute to mouse pre‐implantation embryos. However, most of the core pluripotency factors were not significantly influenced by overexpression of BCL2. Furthermore, RNA‐seq analysis shows BCL2 plays a role in the PI3K signal pathway and Focal adhesion by affecting FGFR2 and BMP5. In addition, BCL2 also improves the anti‐apoptotic ability of cells by affecting intramolecular oxidoreductases activity and lipid metabolism. Our results demonstrate that the overexpression of BCL2 substantially promotes piPSCs survival without compromising their pluripotency.

## 2 MATERIALS AND METHODS

### 2.1 Cell culture

Porcine iPSCs was cultured on feeder (MEF, mouse embryonic fibroblasts) and maintained in LB2i medium, consisting of DMEM (Hyclone) supplemented with 15% FBS (VIS), 0.1 mmol/L NEAA, 1 mmol/L L‐glutaMAX, 10 ng/mL LIF (Sino Biological, 14890 ‐HNAE), 10 ng/mL bFGF (Sino Biological,10014‐HNAE), 0.1 mmol/L β‐mercaptoethanol (Sigma‐Aldrich, M3148), 3 µmol/L CHIR99021 (MCE, HY‐10182), 2 µmol/L SB431542 (Selleck, S1067), 4 µg/mL Dox (Sigma‐Aldrich, D9891), and 100 units/mL penicillin, 100 µg/mL streptomycin. DOX‐piPSCs were passaged using TrypLE™ Select (Invitrogen) into a single cell at a ratio of 1:50 every 5‐6 days.^14^ The KSR medium with different dosage was to replace the FBS in LB2i medium with KSR, and the remaining components were unchanged. HEK293T and MEFs were cultured in DMEM supplemented with 10% FBS and 100 units/mL penicillin, 100 µg/mL streptomycin.

### 2.2 Plasmids and cloning

To clone the porcine BCL2 gene, total RNA was extracted by Trizol (Invitrogen) and was used for reverse transcription (Thermo Fisher). The porcine BCL2 gene was PCR‐amplified from cDNA and subcloned into an in‐house PCDH‐EF1‐GFP‐T2A‐MCS vector using NovoRec^®^ plus One step PCR Cloning Kit (Novoprotein). The primer information used in this study was provided in Table S1. Coding regions of this recombinant vector were verified by sequencing. Subsequently, these overexpression vectors were transfected into HEK293T cell and identified by Western blot.

### 2.3 Lentivirus packaging and transduction

HEK293T cells were seeded onto a 6‐well plate and grown to 70%‐80% confluence. Then the lentivirus backbone and package vector (pVSV‐G and psPAX2) were transfected into HEK293T cells using PEI (polyethyleneimine, sigma). For transfection of per well, 1 µg pVSV‐G, 1 µg psPAX2 and 2 µg lentivirus backbone vector were diluted in 200 µL optiMEM and vortexed. 12 µL PEI (1 mg/mL) was added to the plasmid mix, vortexed, incubated for 15 minutes at room temperature then added to cells. After 12 hours, the media were replaced with fresh media added lipid and cells were maintained for 48‐72 hours. Lentivirus (culture supernate) was collected, filtered through 0.45 µm filter to remove debris. For the lentiviral transduction, 2 × 10^4^ cells were plated on MEF‐coated 12 well plate per well and allowed to attach overnight. Virus and fresh media were added at a ratio of 1:1 supplemented with 4 µg/mL Polybrene into well. The cells were incubated with mixed media overnight, washed with PBS and replaced with fresh medium. After 1 week of culture, stably infected colonies were selected with puromycin (10 µg/mL) for 24 hours, and viral titre was calculated by counting the GFP‐positive colonies. The GFP‐positive colonies were picked up and seeded on 48 well plates per well coated by MEF feeders to continue the growth. After several passages, GFP‐positive colonies were collected and used to detect the expressions of transgene.

### 2.4 AP staining

Cells were fixed with 4% paraformaldehyde in PBS (pH 7.4) for 15 minutes at room temperature, washed twice using ice‐cold PBS and developed with AST Fast Red TR and α‐Naphthol AS‐MX Phosphate (Sigma‐Aldrich) according to the manufacturer's instructions. Then the cells were incubated with the mixture (1.0 mg/mL Fast Red TR, 0.4 mg/mL Naphthol AS‐MX in 0.1 mol/L Tris‐HCL Buffer) at room temperature. After 20 minutes, the AP‐positive iPS colonies showed in red colour. The images were documented by a Nikon phase contrast microscope.^14^

### 2.5 RNA extraction, Reverse transcription and Quantitative real‐time PCR

The method of RNA extraction was described above. Reverse transcription was performed using Fast Quant RT Kit (TIANGEN, KR106). Fluorescence quantitative PCR analyses were performed using a Bio‐Rad CFX96 and SYBR green master mix (TIANGEN, FP215). The primer information used in this study is provided in Table S1. The expression of the target gene was normalized against control vector transfection. Data of Q‐PCR are calculated by Bio‐Rad software CFX3.1 and derived from three independent experiments.

### 2.6 EDU staining

The EDU staining was performed by Cell‐Light EdU Apollo567 In Vitro Kit (RIBOBIO, C10310‐1). The steps are as follows: using 4% paraformaldehyde to fix cells at room temperature for 10 minutes after cells were incubated in the presence of 50 µmol/L EDU medium for 10 minutes at 37°C, and then 2 mg/mL glycine was added to neutralize the excessive aldehyde. Cells were incubated in the presence of 1× Apollo staining solution for 30 minutes at 37°C and then washed three times in PBS. Using 0.1% Triton‐100 to perforate the membrane for 10 minutes at 37°C and nuclei were stained with Hoechst33342.

### 2.7 Apoptosis detection

The apoptosis detection was performed by Annexin V‐FITC/PI Apoptosis Detection Kit (Vazyme, A211). The steps are as follows: the cells were digested by TrypLE™ Select and then transferred immediately to a 1.5 mL tube on ice. The cell suspension was then centrifuged at 1000 *g* for 5 minutes and the supernatant was discarded. The cells were washed twice with pre‐cooled PBS and centrifuged at 1000 *g* for 5 minutes. Add 100 µL 1× Binding Buffer and blow gently to make a single‐cell suspension. Then add 5 µL Annexin V‐FITC and 5 µL PI Staining Solution, and incubate for 10 minutes at room temperature in the dark. Finally, add 400 µL 1× Binding Buffer and mix gently.

### 2.8 Western blot

The cells were digested by TrypLE™ Select and then transferred immediately to a 1.5 mL tube on ice. The cell suspension was then centrifuged at 5000 *g* for 3 minutes and supernatant was discarded. The cell sediment was lysed by RIPA buffer (Beyotime, P0013B) for 30 minutes on ice, added to 5× SDS‐PAGE loading buffer (GENSHARE G, JC‐PE007), and heated at 100°C for 5 minutes, then loaded onto 8%‐12% SDS‐PAGE gel. The SDS‐PAGE gels were run at 100 V for 1.5 hours and transferred to a PVDF membrane by semidry electrophoretic transfer (Bio‐Rad) for 45 minutes at 15 V. The transferred membrane was blocked with 8% skim milk) at room temperature for 2 hours, and then incubated with the anti‐BCL2 antibody (12789‐1‐AP, Proteintech), anti‐BAX antibody (50599‐2‐Ig, Proteintech), anti‐caspase3/cleaved‐caspase3 antibody (WL02117, Wanleibio) in TBS‐T buffer (20 mmol/L Tris/HCl pH 8.0, 150 mmol/L NaCl, 0.05% Tween 20) at 4°C overnight. After washing three times with TBS‐T buffer, the blot was incubated with a secondary antibody at 37°C for 1 hour, then washed three times. Enhanced chemiluminescent substrate (Biodragon, BF06053‐500) was used to detect the HRP signal and the Western blot images were collected using the Chemiluminescent Imaging System (ZY058176, Tanon‐4200). The anti‐β‐ACTIN (KM9001, Sungene Biotech) antibody was used as an internal control.

### 2.9 Immunofluorescence microscopy

The cells were fixed with 4% paraformaldehyde in PBS (pH 7.4) for 15 minutes at room temperature. Fixed cells were washed twice using ice‐cold PBS, permeabilized with 0.1% Triton X‐100 in PBS for 10 minutes, and subsequently blocked for 2 hours at room temperature in PBS containing 5% FBS. The cells were incubated with a blocking buffer containing BCL2 antibodies (12789‐1‐AP, Proteintech) at 4°C overnight. The Alexa Fluor^®^ 594 conjugate goat anti‐rabbit IgG (H + L; #ZF‐0516; ZSGB‐BIO) was diluted in blocking buffer and incubated at 37°C for 1 hour. After washing with PBS for three times, the nuclei were stained by 10 µg/mL Hoechst 33342 for 8 minutes. Finally, the images were collected by an EVOS fluorescence microscope.^21^

### 2.10 Digital RNA‐seq

#### 2.10.1 RNA extraction, library preparation and sequencing

Total RNAs were extracted from samples using TRIzol (Invitrogen) following the methods.^22^ DNA digestion was carried out after RNA extraction by DNase I. RNA quality was determined by examining A260/A280 with Nanodrop™ One spectrophotometer (Thermo Fisher Scientific Inc). RNA integrity was confirmed by 1.5% agarose gel electrophoresis. Qualified RNAs were finally quantified by Qubit3.0 with Qubit™ RNA Broad Range Assay kit (Life Technologies).

Two Microgram total RNAs were used for stranded RNA sequencing library preparation using KC‐Digital™ Stranded mRNA Library Prep Kit for Illumina^®^ (Catalog NO. DR08502, Wuhan Seqhealth Co., Ltd.) following the manufacturer's instruction. The kit eliminates duplication bias in PCR and sequencing steps, by using the unique molecular identifier (UMI) of 8 random bases to label the pre‐amplified cDNA molecules. The library products corresponding to 200‐500 bps were enriched, quantified and finally sequenced on Hiseq X 10 sequencer (Illumina).

#### 2.10.2 RNA‐Seq data analysis

Raw sequencing data were first filtered by Trimmomatic (version 0.36), low‐quality reads were discarded and the reads contaminated with adaptor sequences were trimmed.^23^ Clean Reads were further treated with in‐house scripts to eliminate duplication bias introduced in library preparation and sequencing. Briefly, clean reads were first clustered according to the UMI sequences, in which reads with the same UMI sequence were grouped into the same cluster, resulting in 65 536 clusters. Reads in the same cluster were compared to each other by pairwise alignment, and then reads with sequence identity over 95% were extracted to a new sub‐cluster. After all sub‐clusters were generated, multiple sequence alignment was performed to get one consensus sequence for each sub‐cluster. After these steps, any errors and biases introduced by PCR amplification or sequencing were eliminated.^24^, ^25^

The de‐duplicated consensus sequences were used for standard RNA‐seq analysis. They were mapped to the reference genome of Sus scrofa from GCF_000003025.6 (ftp://ftp.ncbi.nlm.nih.gov/genomes/all/GCF/000/003/025/GCF_000003025.6_Sscrofa11.1/GCF_000003025.6_Sscrofa11.1_genomic.fna.gz) using STRA software (version 2.5.3a) with default parameters.^26^ Reads mapped to the exon regions of each gene were counted by feature Counts (Subread‐1.5.1; Bioconductor) and then FPKMs were calculated.^27^ Genes expressed differentially between groups were identified using the edgeR package (version 3.12.1).^28^ An FDR corrected p‐value cut‐off of 0.05 and a fold‐change cut‐off of 2 were used to judge the statistical significance of gene expression differences. Gene ontology (GO) analysis and Kyoto encyclopedia of genes and genomes (KEGG) enrichment analysis for differentially expressed genes were both implemented by KOBAS software (version: 2.1.1) with a corrected P‐value cut‐off of 0.05 to judge statistically significant enrichment.^29^

### 2.11 Embryoid body formation and spontaneous differentiation

2 × 10^6^ NC‐piPSCs and BCL2‐piPSCs were digested into single cells and were suspended in a 35 mm anti‐adherent dish in LB2i medium without DOX. After 2 days in suspension culture, the medium was replaced with DMEM supplemented with 15% FBS. On the fifth day of suspension culture, EBs were transferred to gelatin‐treated petri dishes allowing the spontaneous differentiation for another 5 days in the same medium.^14^, ^30^

### 2.12 Interspecific chimeric embryo formation assay

Select 5‐6 weeks old KunMing female mice. Inject 100 µL PMSG solution (100 IU/mL) into a female mouse. After 48 hours, inject 100 µL HCG solution (100 IU/mL). After 2 hours, cross the female mice with normal 5‐6 weeks oestrus male mice.

Humanely kill the 1.5 days pregnant mice by cervical dislocation. Cut the skin and the peritoneum and locate the bilateral oviduct. Remove the fat pads and pick off the oviduct carefully, then transfer them to a 35 mm Petri dish containing the prewarmed M2 culture medium (Sigma‐Aldrich, M7167). Strip the oviduct under a stereomicroscope and collect the 2‐cell stage embryos. Wash the embryos three times with prewarmed M2 medium to remove tissue impurities. Transfer the embryos into 10 µL drops of prewarmed KSOM medium (caisson, IVL04) covered with mineral oil. Keep the plate in a 37°C, 5% CO_2_ incubator.

Culture the E1.5 embryos in KSOM medium until E2.5, and then select the well‐developed 8/16‐cell stage embryos, continue the microinjection using porcine iPSCs. Inject 8‐10 single porcine iPSCs for each embryo. Passage the injected embryos through 3‐5 drops of KSOM to wash away any residual M2 and impurities. Then culture the injected embryos in 37°C, 5% CO_2_ incubator and observing the location and survival numbers of piPSCs.^30^

## 3 RESULTS

### 3.1 The gene cloning and overexpression of porcine BCL2 in piPSCs

**FIGURE 1:**
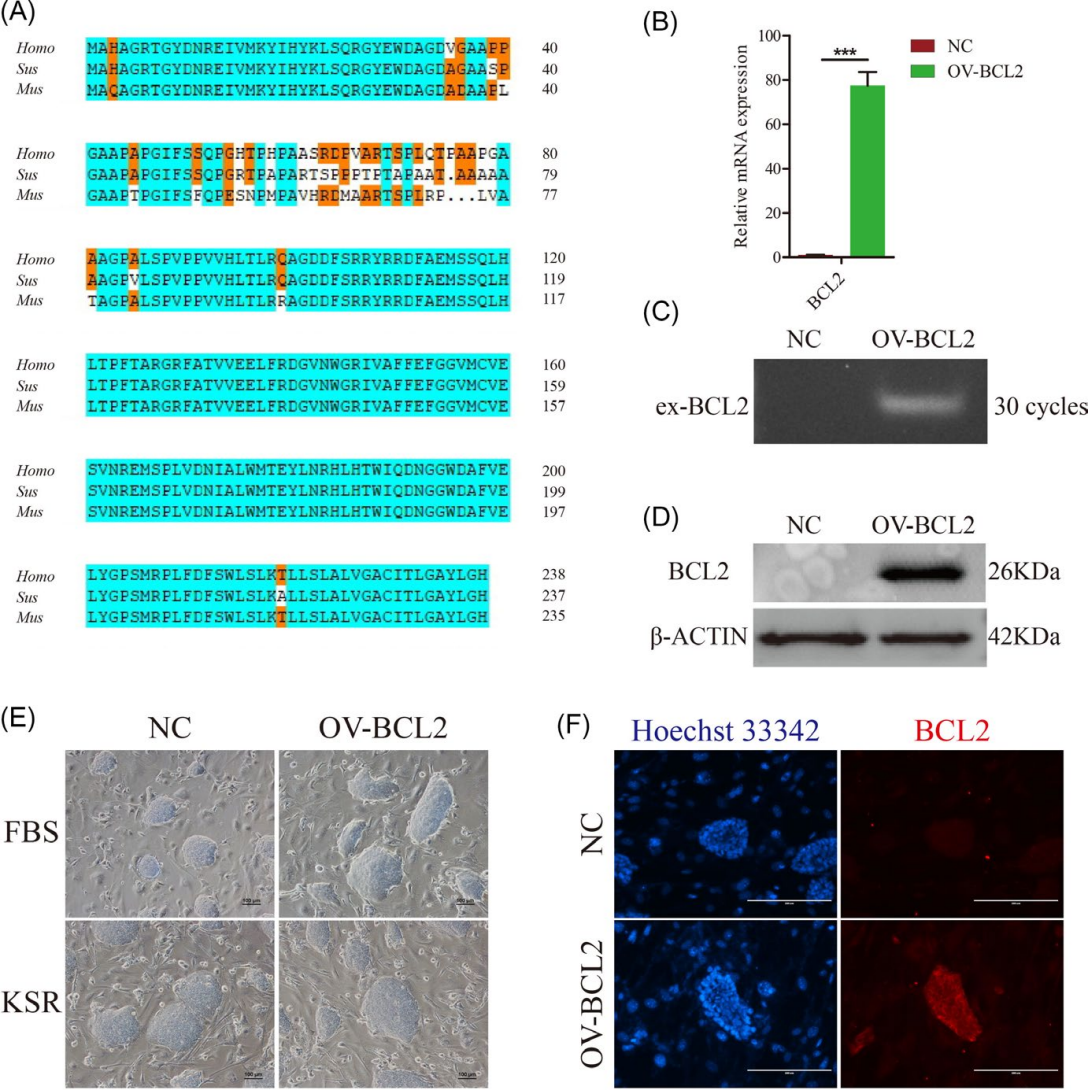
Clone and overexpression of BCL2 gene in piPSCs. A, Sequence alignment of BCL2 proteins among human, mouse and pig. The blue region represents the consistency of amino acid sequences between species, and the orange region represents the consistency between only two species. B, RT‐qPCR analysis of the BCL2 in the control (NC) and overexpressed BCL2 (OV‐BCL2) groups. The relative expression levels were normalized to β‐actin. Data represent the mean ± SD; n = 3 independent experiments. C, Semi‐quantitative RT–PCR analysis of the exogenous BCL2 (ex‐BCL2) in the control (NC) and overexpressed BCL2 (OV‐BCL2) groups. D, Representative Western Blot of BCL2 after 5 d of culture in the indicated conditions. Data represent the mean ± SD; n = 3 independent experiments. E, Representative image of NC‐piPSCs (NC) and BCL2‐piPSCs (OV‐BCL2) cultured in LB2i and 20% KSR medium. The experiments were performed three times. The scale bar represents 100 µm. F, Immunofluorescence analysis of BCL2 in the indicated conditions. The nuclei were DAPI stained. The scale bar represents 200 µm. The experiments were performed three times

To determine whether overexpression of porcine BCL2 could improve the survival of piPSCs, we first obtained the coding sequence of the BCL2 gene according to the sequencing data of piPSCs in our laboratory. Further, the coding sequence of the porcine BCL2 was cloned and inserted into the lentiviral vector. The coding sequence of porcine BCL2 was validated again by the dideoxy chain‐termination method and translated into protein sequences according to the codon. By comparing the sequence of BCL2 protein with that of mouse and human, it can be seen that this protein is highly conserved, with only significant differences in the position 50‐80 of N‐terminal (Figure 1A). Previous studies demonstrated that this region belongs to the loop of BCL2 protein associated with anti‐apoptosis.^31^ This result suggests that the role of porcine BCL2 in piPSCs may be different from that of other species.

To further investigate the role of porcine BCL2 in piPSCs, constitutive BCL2‐expressing piPSCs lines were generated using lentiviral vector. Semi‐quantitative PCR analysis showed that the exogenous BCL2 gene had been overexpressed in piPSCs and the expression of total BCL2 in BCL2‐expressing piPSCs lines was about 77 times higher than that of the NC group (Figure 1B,C). In addition, both Western blot and fluorescence analysis indicated that BCL2 protein had been significantly up‐regulated in BCL2‐expressing piPSCs lines, and the expression of BCL2 protein in the NC group could not be detected by Western blot (Figure 1D,F). This result suggested that BCL2 was not indispensable for the maintenance of pluripotency. Compared with the NC group, there was no significant change in the clone morphology of piPSCs when the BCL2 was overexpressed, which remained the dome‐shaped morphology under the two media (Figure 1E). Further observation revealed that the BCL2‐expressing piPSCs exhibited fewer dead cells than the NC group (Figure 1E). These results indicated that BCL2 may not significantly influence the pluripotency of piPSCs but has a certain inhibitory effect on cell death.

### 3.2 BCL2 enhanced the pluripotency and anti‐apoptotic ability of piPSCs

**FIGURE 2:**
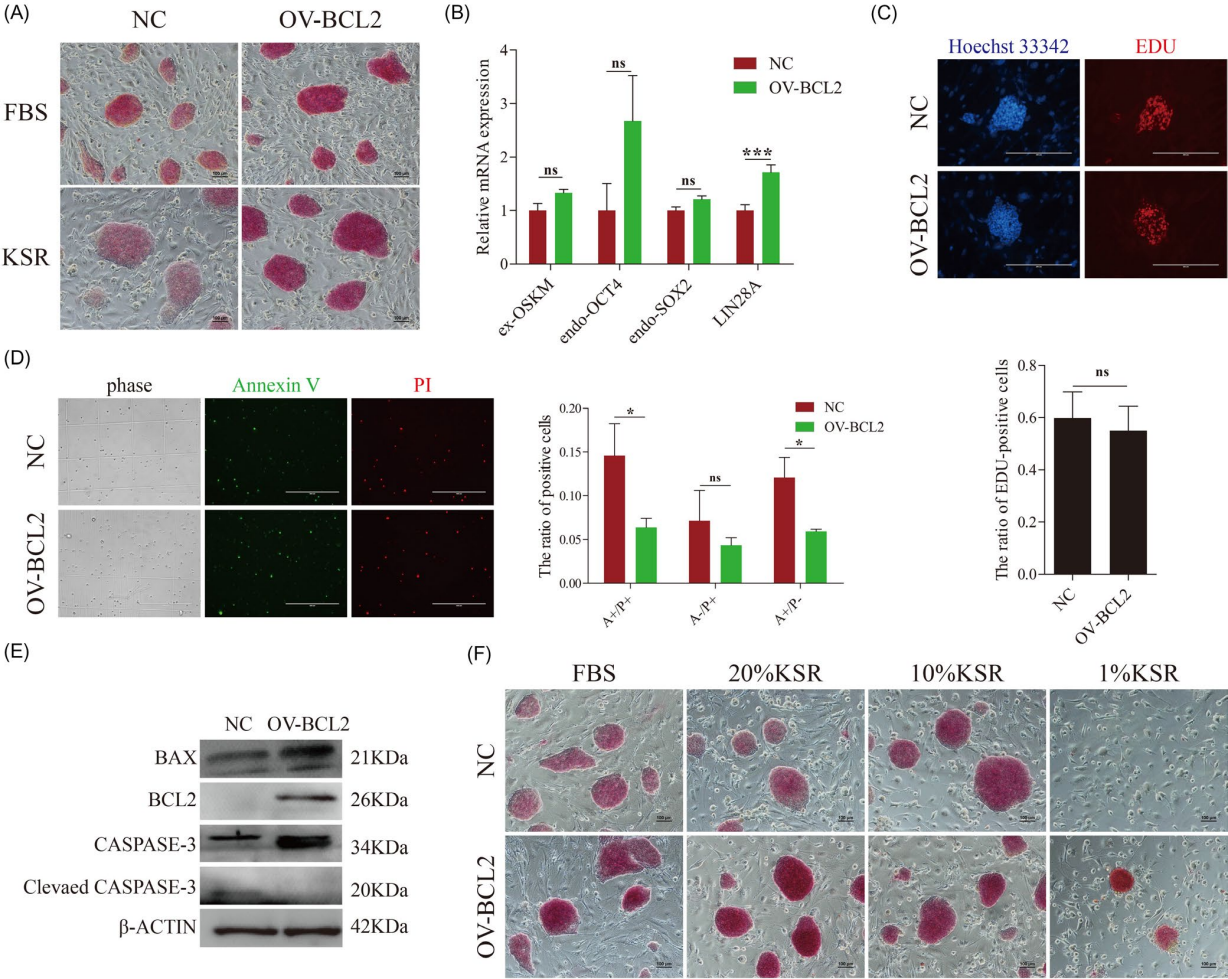
Overexpression of BCL2 enhances the pluripotency and inhibited apoptosis in piPSCs. A, Representative image of AP staining of NC‐piPSCs (NC) and BCL2‐piPSCs (OV‐BCL2) cultured in LB2i and 20% KSR medium. The experiments were performed three times. The scale bar represents 100 µm. B, RT–qPCR analysis of the ex‐OSKM, endo‐OCT4, endo‐SOX2 and LIN28A in the control (NC) and overexpressed BCL2 (OV‐BCL2) groups. The relative expression levels were normalized to β‐actin. Data represent the mean ± SD; n = 3 independent experiments. C, Representative images of NC and BCL2‐piPSCs cultured in LB2i medium and exposed to EDU in order to evaluate proliferation. The quantitative analysis is shown below. n = 3 independent experiments. The scale bar represents 200 µm. D, Representative images of cells stained with PI (P) and Annexin V (A) after 5 d of culture in LB2i medium. A+/P+: non‐viable apoptotic cell or necrotic cells; A−/P+: mechanic injury; A+/P−: viable apoptotic cell. The quantitative analysis is shown on the right. n = 3 independent experiments. The scale bar represents 400 µm. n = 3 independent experiments. E, Representative Western Blot of BAX, BCL2 and CASPASE3 after 5 d of culture in the indicated conditions. Data represent the mean ± SD; n = 3 independent experiments. F, Representative image of AP staining of NC‐piPSCs (NC) and BCL2‐piPSCs (OV‐BCL2) cultured in LB2i and KSR (20%, 10%, 1%) medium. The experiments were performed three times. The scale bar represents 100 µm

In previous reports, overexpression of BCL2 in ESCs provides a survival benefit under conditions of stress by resisting apoptosis.^15^ We further investigated the role of BCL2 on the pluripotency and cell viability of piPSCs. Alkaline phosphatase staining was used to preliminarily determine the effect of BCL2 on the pluripotency of piPSCs. The result showed that the AP staining was stronger after overexpressing BCL2 in both pluripotent media, and the difference was more obvious after the elimination of unknown factors from the serum (Figure 2A). Further analysis of several major pluripotent genes revealed no significant differences in the expression levels of exogenous OSKM, OCT4 and SOX2 between the two groups, while the expression of LIN28A was slightly up‐regulated (Figure 2B). These results demonstrated that overexpression of BCL2 slightly enhanced the pluripotency of piPSCs, but it could not influence the expression of core pluripotent transcription factors.

We further detected the proliferation and death of piPSCs after overexpression of BCL2. The results showed that the overexpression of BCL2 significantly inhibited the apoptosis of piPSCs at the late stage of culture, while did not influence the proliferation of piPSCs (Figure 2C,D). The Western blotting analysis further found that the expression levels of pro‐apoptotic protein BAX were significantly increased when the BCL2 was overexpressed (Figure 2E). However, the ratio of BCL2 to BAX suggested that the apoptosis of the BCL2 group was inhibited (Figure 2E). The detection of caspase‐3 showed that the expression of cleaved caspase‐3 in the NC group was significantly higher than that in the BCL2 group, which also indicated that more severe apoptosis occurred in the NC group (Figure 2E). To further investigate the anti‐apoptotic effect of BCL2 in piPSCs, we induced cell apoptosis by reducing the content of KSR in the KSR medium. The results showed that the BCL2‐piPSCs exhibit more compact with a strong AP activity under each culture condition. In addition, BCL2‐piPSCs can survive at 1%KSR medium compared to the NC group, but growth was inhibited (Figure 2F). These results suggested that BCL2 also had a significant anti‐apoptotic effect in piPSCs.

### 3.3 BCL2 enhances survival of piPSCs by the PI3K signalling pathway

**FIGURE 3:**
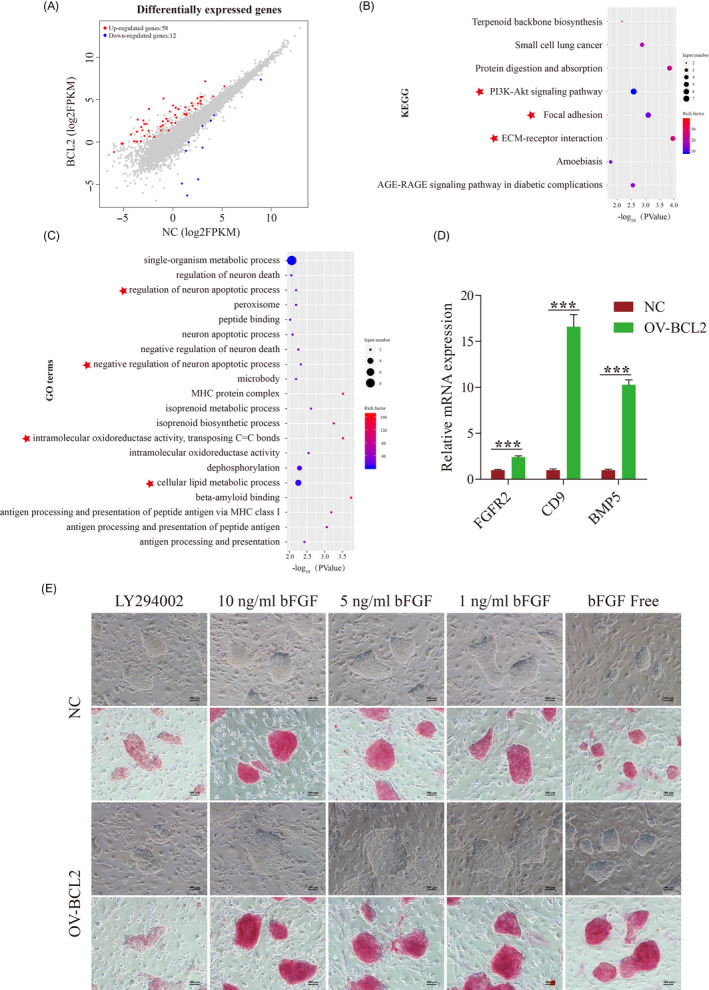
BCL2 enhances pluripotency and inhibits apoptosis by promoting expression of FGFR2. A, Transcriptional changes after overexpression of BCL2 in piPSCs. B, KEGG enrichment of up‐differentially expressed (DE) genes in OV‐BCL2 cell lines. C, Gene ontology enrichment of up‐differentially expressed (DE) genes in OV‐BCL2 cell lines. D, RT‐qPCR analysis of the FGFR2, CD9 and BMP5 in the control (NC) and overexpressed BCL2 (OV‐BCL2) groups. The relative expression levels were normalized to β‐actin. Data represent the mean ± SD; n = 3 independent experiments. E, Representative image of AP stained colonies after 5 d of clonal growth of NC and OV‐BCL2 cell lines. 10 ng/mL bFGF, 5 ng/mL bFGF, 1 ng/mL bFGF and bFGF free represents the concentration of bFGF in the medium. 10 ng/mL bFGF was used as a control. LY294002 is a PI3K inhibitor. The final concentration of this treatment is 10 µmol/L. The experiments were performed three times. The scale bar represents 100 µm

We attempted to gain insights into the molecular consequences that were triggered in response to the overexpression of BCL2. RNA sequencing was performed on these two cell lines after 5 days of culture. Transcriptional changes in both directions were observed after overexpression of BCL2 (58 up vs. 12 down; Table S2), indicating that BCL2 mainly promoted the expression of some genes to improve the anti‐apoptotic ability of piPSCs (Figure 3A). To further investigate this process, we applied KEGG and GO analysis for the up‐regulated genes. KEGG analysis of up‐regulated genes revealed that these genes were mainly enriched in PI3K‐AKT and the Focal adhesion signalling pathway (Figure 3B). GO enrichment revealed that the up‐regulated gene was related to the regulation of neuron death, oxidoreductase activity, and lipid metabolism (Figure 3C). These data suggested that in addition to the classical role of BCL2, BCL2 may indirectly resist apoptosis by promoting the metabolism of these cells and reducing oxidative damage.

Further specific to up‐regulated genes, the Q‐PCR analysis again showed that the expression of BMP5, FGFR2 and CD9 genes was significantly up‐regulated in the BCL2 group vs. the NC group (Figure 3D). Previous studies demonstrated that these three genes play a role in the maintenance of pluripotency and survival.^32^, ^33^, ^34^, ^35^ The increased expression of FGFR2, which is one of the receptors of FGF2, can significantly amplify the effect of FGF2. This result suggested that BCL2 could indirectly promote the expression of FGFR2 in piPSCs, and then enhanced the effect of the FGF signalling pathway, so as to contribute to the survival of piPSCs under stress. To verify this inference, we reduced the concentration of FGF2 in the culture medium. The results showed that the proliferation of piPSCs was significantly inhibited and severe cell death occurred in the NC group when FGF2 was removed. However, this phenomenon was significantly alleviated after overexpression of BCL2 (Figure 3E). The up‐regulated genes were mainly receptors of the PI3K signalling pathway, such as FGFR2. To further demonstrate the importance of activation of this pathway after overexpression of BCL2, LY294002, an inhibitor of the PI3K signalling pathway, was added to medium. The results showed that the effect of overexpressing BCL2 was abolished (Figure 3E). These results suggested that BCL2 indirectly activates the PI3K signalling pathway, thereby enhancing pluripotency and anti‐apoptosis ability of piPSCs.

### 3.4 BCL2 enhanced survival during embryoid body formation and interspecific chimeric embryos

**FIGURE 4:**
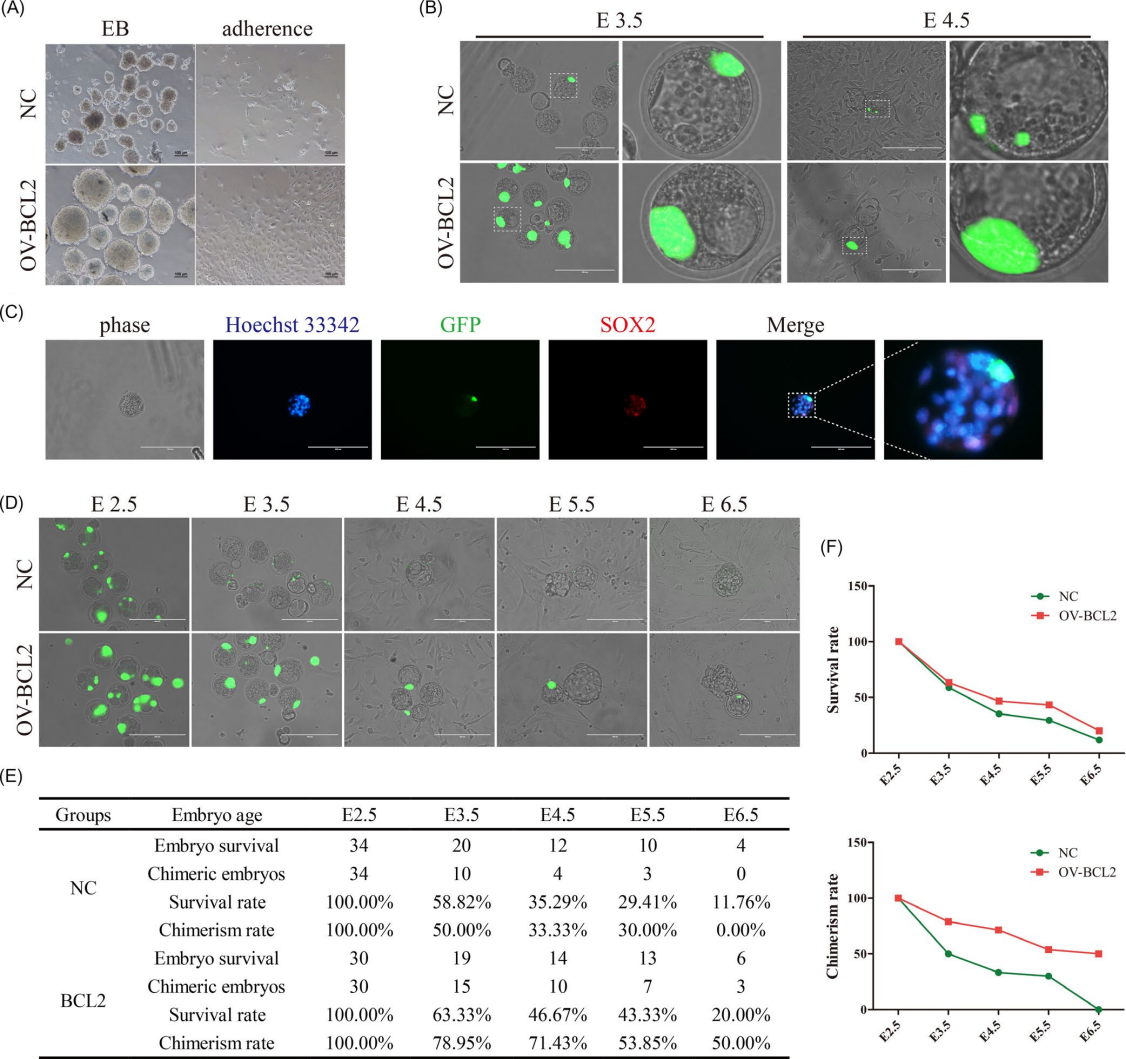
BCL2 overexpression enables piPSCs to form chimeras with pre‐implantation embryos. A, Representative image of NC‐piPSCs (NC) and BCL2‐piPSCs (OV‐BCL2) after suspension culture for 5 d (EB) and continued adherent culture for another 5 d. The experiments were performed three times. The scale bar represents 100 µm. B, Localization of injected (NC) and BCL2‐piPSCs in early (E3.5) and late (E4.5) blastocysts. The dotted white box indicates that the area has been enlarged to the right. The scale bar represents 200 µm. C, Localization of BCL2‐piPSCs in late (E4.5) blastocysts. ICM was indicated by Immunofluorescence of SOX2. The dotted white box indicates that the area has been enlarged to the right. The nuclei were DAPI stained. The scale bar represents 200 µm. D, Representative images of chimeric embryos at different embryonic ages. The scale bar represents 200 µm. E, Statistical table of embryo survival rate and chimerism rate at different embryo ages. F, The line chart exhibits the embryo survival rate and chimerism rate at different embryo ages

To investigate the role of BCL2 in the differentiation and development of piPSCs, BCL2‐piPSCs were used to perform embryoid body potentiality. The results showed that the number and diameter of embryoids formed by NC‐iPSCs were less and smaller than those of BCL2‐piPSCs, and there are a large number of dead cells at the edges of embryoid in the NC‐iPSCs (Figure 4A). After 5 days in adherent culture, the cell survival rate of the BCL2 group was significantly higher than that of the NC group (Figure 4A). These results indicated the overexpression of BCL2 could significantly enhance the survival of piPSCs during suspension differentiation.

E2.5, the well‐developed 8/16‐cell mouse embryos were selected as recipient embryos. The BCL2‐piPSCs and NC‐piPSCs were used as donor cells. Every 8‐10 donor cells carrying GFP tag were injected into one embryo. E3.5, in vitro, the injected embryos developed into early chimera blastocysts. The results showed GFP signal of the BCL2 group is significantly stronger than the NC group in the chimera blastocysts (Figure 4B). At the same time, we observed that BCL2‐piPSCs can locate in the region of ICM (Figure 4B,C). These results suggested overexpression of BCL2 increased the survival rate of piPSCs in mouse embryos.

E4.5, most of the injected embryos started to hatch naturally. The GFP signal of the NC group was very weak, indicating that most of the injected cells had died during embryonic development (Figure 4B). However, the GFP intensity of BCL2 group was remarkably higher than the control. BCL2‐iPSCs were still located in the ICM, although there were no obvious migration phenomena (Figure 4B). E5.5, the statistics showed that the survival rate of BCL2 group was 43.33%, which was significantly higher than that of NC group (29.4%) (Figure 4D‐F). E6.5, There were no GFP‐donor cells survived in the NC group, while the BCL2 group still showed obvious GFP fluorescence, indicating that the cells derived from BCL2‐piPSCs can survive in receptor embryos (Figure 4D‐F). However, BCL2‐iPSCs only exist in the form of original clone‐like, instead of diffusing into the mouse embryonic cells and developing synchronously (Figure 4D‐F). These results suggested overexpression of BCL2 improves the survival of piPSCs in embryos, but it may be unable to participate in the next stage of mouse embryonic development due to interspecific differences and even interrupt the development process of mouse embryos.

## 4 DISCUSSION

The generation of chimeras from pluripotent stem cells of domestic animals, especially pigs, is a challenging issue across the globe. Porcine pluripotent stem cells (pPSCs) have been obtained by reprogramming or isolating ICM.^4^, ^9^, ^36^ However, the majority of these pPSCs have had limited ability to survive extended passage and failed to contribute to chimeras and exhibited limited differentiation potential.^4^ In our study, the piPSCs generated by TetO‐inducible system exhibited similar characteristics to those pPSCs in previous studies.^14^ Our results demonstrated that porcine BCL2 gene could enhance the survival of piPSCs under stress and contribute to forming chimeras.

BCL2, a 26‐kDa anti‐apoptotic protein, can enhance the survival of mouse and human pluripotent stem cells through anti‐apoptotic effect, and the overexpression of it can overcome stage‐related compatibility barriers to form chimeras.^15^, ^16^, ^17^ In addition, it has been reported that overexpression of BCL2 in PSCs can also contribute to form interspecific chimera.^37^ However, so far there is no coding sequence of porcine BCL2 gene and no report on its role of pPSCs. Previous studies have demonstrated that BCL2 protein contains four major domains: BH1, BH2, BH3 and BH4.^38^, ^39^ In this study, the results indicated that there were no significant differences in the protein sequences of these major domains. The differences among different species mainly located in the N‐terminal loop region between BH3 and BH4, which was previously reported to interact with Nur77 and influence the anti‐apoptotic effect of BCL2.^31^ However, our results showed that the enforced expression of BCL2 in piPSCs could still enhance survival in several different systems, which was consistent with the studies on human and mouse pluripotent stem cells suggesting that this loop region of BCL2 was not the core of the anti‐apoptotic mechanism.

Previous studies of BCL2 in pluripotent stem cells have focused more on anti‐apoptotic effects rather than on molecular mechanisms. The viability of pluripotent stem cells depends on the addition of cytokines, such as FGF2, which can directly inhibit pro‐apoptotic BCL2 family members, while the overexpression of BCL2 also plays the same role, which is equivalent to the replacement of cytokines.^15^ In this study, we found that BCL2 was mainly located in the cytoplasm of piPSCs (Figure 1E), and after the serum in the medium was replaced by KSR, the effect of BCL2 on the maintenance of pluripotency was more obvious, which was similar to studies in other species.^15^ In addition, we also found that the expression of BCL2 may not alone inhibit apoptosis through interaction with BAX. FGF2 is a key cytokine to maintain the survival, self‐renewal and proliferation of piPSCs by activating the FGF signalling pathway.^32^, ^40^ We found that BCL2 can further enhance the expression of FGFR2, indicating that BCL2 indirectly enhances the effect of FGF2. Combined with previous studies, there may be a feedback regulation mechanism that needs to be further explored.

The PI3K signalling pathway plays an important role after the overexpression of BCL2. PI3K/AKT/BCL2 is a common apoptotic inhibitory approach in other previous studies.^41^, ^42^, ^43^ Activated AKT directly promotes the expression of BCL2 to play an anti‐apoptotic role.^43^ Therefore, it is traditionally believed that BCL2 is an important anti‐apoptotic gene in the downstream of the PI3K signalling pathway. However, in our study, overexpression of BCL2 could further activate this pathway by increasing the expression of the receptor of this signalling pathway, indicating that there is a novel positive feedback regulation mechanism in piPSCs. We abolished the effect of BCL2 by inhibiting the PI3K signalling pathway, and further demonstrated the importance of this regulatory mechanism for the pluripotency of piPSCs. However, the mechanism by which BCL2 activates FGFR2 and other receptors needs further research.

Oxidative stress is another major cause of apoptosis in cell culture and differentiation. In our study, although there were only 58 up‐regulated genes after the overexpression of BCL2, some of which were related to oxidoreductase activity, indicating that the anti‐apoptotic mechanism of BCL2 on piPSCs was similar to that on other types of cells. Previous studies on mouse and human pluripotent stem cells showed that BCL2 substantially promoted the survival of pluripotent stem cells without compromising their pluripotency.^15^, ^17^ However, in this study, overexpression of BCL2 slightly increased pluripotency of piPSCs. The overexpression of BCL2 did not influence the expression of core pluripotent genes but increased the expression of genes related to lipid metabolism. Previous studies have demonstrated that lipid metabolism is more important in porcine embryonic development than in mouse.^44^, ^45^ Therefore, it was speculated that BCL2 enhanced the pluripotency of piPSCs by promoting lipid metabolism.

In the last part of this study, the BCL2‐piPSCs were injected into 8/16‐cell mouse embryos to evaluate whether BCL2 can improve the chimeric ability of pPSCs. Previous studies have demonstrated that overexpression of BCL2 in pluripotent stem cells is beneficial for the formation of interspecific chimera.^37^ This effect may be attributed to BCL2 helps stem cells overcome interspecies compatibility barriers to form chimeras. However, BCL2‐iPSCs were not diffusing into the mouse early embryo and developing synchronously, suggesting that it did not participate in the next stage of mouse embryonic development. Besides, the presence of BCL2‐iPSCs also significantly influenced the hatching process of embryos. Recent studies have shown that porcine early embryo has a unique signalling regulatory network at this developmental stage,^46^ so it may be that the incompatibility of pPSCs and mouse early embryo in developmental signals leads to mutual perturbations, thus delaying the development of chimeric embryos. This may be the reason why chimeric embryos were transplanted in vivo and did not develop into adult chimeras.

In summary, overexpression of BCL2 enables piPSCs to enhance survival under stress and contribute to forming chimeras, and this effect was mediated mainly by increased resistance to apoptosis. A series of signalling pathways that interact with the BCL2 pathway, such as the PI3K signal pathway, may impact the survival of piPSCs. We found that the BCL2 promoted the expression of FGFR2 and thus activated the FGF signal pathway to improve the survival of piPSCs. However, the activation mechanism of this process still needs to be further studied.

## CONFLICT OF INTEREST

All authors read and approved the final manuscript and declare no competing financial interests.

## AUTHOR CONTRIBUTIONS

Zhenshuo Zhu and Jinlian Hua designed the study and wrote the manuscript. Zhenshuo Zhu, Qin Pan, Wenxu Zhao, Xiaolong Wu, Juqing Zhang, Shuai Yu, Qiaoyan Shen and Wei Yue performed the experiments. Zhenshuo Zhu, Sha Peng, Na Li, Shiqiang Zhang, Anmin Lei and Jinlian Hua analysed the data.

## Supporting information

Table S1‐S2

## Data Availability

The data that support the findings of this study are available from the corresponding author upon reasonable request.
