# Supplementary material for: BCL2 enhances survival of porcine pluripotent stem cells through promoting FGFR2
Source: Cell Prolif. 2020 Oct 26;54(1):e12932. doi: 10.1111/cpr.12932 (PMC7791183; doi:10.1111/cpr.12932)
Supplement: Supplementary file 1 — Table S1‐S2 [file CPR-54-e12932-s001.docx]

**Supplementary table 1**

**the information of primers used in this experiment.**

| primer name | forward sequence | reverse sequence |
| --- | --- | --- |
| BCL2 | TGTGTGGAGAGCGTCAACCG | CCCATACAGCTCCACAAAGGCAT |
| FGFR2 | GGTCCATCAATCACACATACCACC | TGGGGCTGGGCATCACTGTA |
| CD9 | GACTGCTGTGGTTTAATGGGG | GTGGAACTTGTTTTGGAAGACCTC |
| BMP5 | GATGTGGGTTGGCTTGTCTTTG | CTTCACGCTGATGCTGTGTCC |
| ex-BCL2 | CAGTCGTCCAATTCTGCCGTG | ATACCCTGTTCTCCCAGCGTG |
| ex-OSKM | TCGGACCACCTTGCCTTACAC | CAACGCCCAAAGGAAATCCAG |

**Supplementary table 2**

**the FPKM of differentially expressed genes.**

| Gene names | NC-rep1 | NC-rep2 | BCL2-rep1 | BCL2-rep2 |
| --- | --- | --- | --- | --- |
| MLKL | 0.11026399 | 0.098312981 | 1.069283763 | 1.082545801 |
| APOE | 11.1934106 | 8.234494882 | 103.4429879 | 182.0992583 |
| SCG5 | 9.50059329 | 4.658977003 | 40.20300704 | 40.15988782 |
| SLC2A5 | 8.62535576 | 7.183427294 | 51.54652207 | 29.72648305 |
| EMILIN2 | 2.21711954 | 0.770188074 | 15.7699879 | 13.97746627 |
| DSC3 | 0 | 0 | 5.078161197 | 3.930204749 |
| DSEL | 0.66037809 | 0.814301633 | 2.663175778 | 2.461943016 |
| LOC100525112 | 1.86824631 | 1.88069181 | 0.066412377 | 0 |
| BCL2 | 1.32173968 | 1.04148202 | 4.411088337 | 5.118262668 |
| BMP5 | 0.61082049 | 0.635385849 | 6.731164198 | 6.746502525 |
| HAPLN3 | 0.14002944 | 0.166469715 | 3.425696704 | 5.308162566 |
| ISLR2 | 0.61465438 | 0.827760878 | 0 | 0 |
| THTPA | 0.16388268 | 0.194826908 | 2.131040213 | 2.905069145 |
| NDRG2 | 0 | 0 | 3.815879511 | 7.185191461 |
| SLIT2 | 0.155876 | 0.231635511 | 1.223885235 | 1.939510717 |
| C1H14orf39 | 2.27371918 | 2.762398827 | 0.558683232 | 0.41897217 |
| SIX6 | 6.20642631 | 9.765423615 | 0.649898016 | 0.61270212 |
| INPP4B | 0.04261886 | 0.010555444 | 0.900171028 | 0.852340935 |
| LOC106507797 | 0.96110019 | 0.432793394 | 5.744924707 | 5.281854517 |
| PFKFB2 | 1.35714046 | 1.743286888 | 0 | 0 |
| COL1A2 | 1.84432953 | 2.633937191 | 8.340872145 | 11.39864668 |
| PEG10 | 450.160383 | 530.8831636 | 176.439532 | 151.4800788 |
| DGKB | 3.28510517 | 2.741169672 | 0.970701115 | 1.017425417 |
| CACNA2D1 | 9.06365164 | 7.037925508 | 3.61538512 | 3.829548793 |
| LPGAT1 | 18.0280566 | 18.44804421 | 9.571712403 | 8.092666997 |
| FRMD3 | 0.11651557 | 0.038476658 | 1.084255471 | 0.953268568 |
| COL4A1 | 6.71565747 | 7.541424896 | 19.51059153 | 26.22324763 |
| COL4A2 | 2.1753072 | 2.795726945 | 7.990536319 | 10.90279515 |
| ITGA3 | 0 | 0 | 1.402074402 | 1.228887774 |
| COL1A1 | 0.71412277 | 0.265300941 | 5.049622909 | 5.815524787 |
| CA10 | 0.0231863 | 0.034455396 | 0.97093808 | 0.75879186 |
| ABCA1 | 7.02408339 | 6.784672917 | 33.2452355 | 24.43957614 |
| PMP22 | 0.18151655 | 0.0674345 | 6.150889693 | 5.507142542 |
| LOC100517188 | 4.98032307 | 2.500029477 | 21.30560312 | 26.83652244 |
| FUCA2 | 0 | 0 | 4.132273259 | 4.670708701 |
| IMPDH2 | 41.21169 | 34.52152258 | 86.9244266 | 103.6213891 |
| KLHDC8B | 0.21368001 | 0 | 3.814657033 | 3.030241949 |
| NUP210 | 0.92297299 | 1.086896811 | 19.76637068 | 19.59057319 |
| SERPINI1 | 2.54375905 | 2.700062618 | 11.67995896 | 8.671536388 |
| LOC100153543 | 0.53361492 | 0.29736177 | 3.675230397 | 3.395586647 |
| EHHADH | 0.48321104 | 0.380151173 | 2.568492872 | 2.674342874 |
| EPHX2 | 4.23516855 | 3.689330165 | 17.54186587 | 21.4072675 |
| CCDC60 | 0.5642903 | 0.397207456 | 2.847376013 | 4.940704545 |
| CYP2C91 | 0.16764984 | 0 | 7.944163533 | 4.572073457 |
| FGFR2 | 5.11135511 | 4.653158953 | 13.75178118 | 16.70224241 |
| UNC5D | 0.32885683 | 0.211765329 | 1.340865356 | 1.629266668 |
| SEMA5A | 0.15866811 | 0.073682705 | 2.436975195 | 2.866722072 |
| ZC3HAV1L | 0 | 0 | 2.120694107 | 1.142756204 |
| PLP2 | 0.85088148 | 0.180632932 | 30.54094453 | 40.11127499 |
| HEPH | 3.98187349 | 2.216165024 | 16.1549754 | 12.03865513 |
| ARMCX2 | 5.45476281 | 6.291999976 | 0.04203552 | 0.052013958 |
| GPRASP2 | 1.21038551 | 1.341375222 | 6.284823501 | 8.504041229 |
| BHLHB9 | 1.49460457 | 0.941109596 | 16.16327803 | 17.37490139 |
| LOC110258909 | 0.36852528 | 0 | 16.04135206 | 14.32162238 |
| LOC110259040 | 0.10628063 | 0.1579356 | 4.567676397 | 4.202736895 |
| TRIM44 | 0.06356869 | 0.094464612 | 1.611193384 | 3.033830841 |
| SPON1 | 4.37636506 | 7.303801226 | 23.05002653 | 23.99247569 |
| SWAP70 | 2.89375975 | 1.929575666 | 15.16765234 | 19.22345902 |
| COL5A3 | 1.16692317 | 0.623183614 | 5.183923479 | 4.425500174 |
| EFNA2 | 12.8182354 | 16.07871623 | 5.854308179 | 5.648998923 |
| CNN2 | 17.7946444 | 16.22415973 | 46.71898116 | 37.02494626 |
| ZBED3 | 2.17779292 | 3.269964082 | 0.02499896 | 0 |
| LOC106509501 | 0.01802807 | 0.013395071 | 0.278133777 | 0.602275596 |
| FBXL21 | 0.17325406 | 0.096547403 | 1.384197372 | 1.889963793 |
| NIPAL2 | 0.06886408 | 0.034111232 | 1.745408891 | 1.909331826 |
| LYN | 0.09918372 | 0.029477882 | 1.092991972 | 1.054909147 |
| CD9 | 0.70519706 | 0.898234231 | 13.65507687 | 10.57750518 |
| B2M | 1.25349166 | 1.086586091 | 11.62620997 | 6.694499753 |
| B2M | 6.39792872 | 4.929803045 | 43.60825322 | 27.46468543 |
| C5H12orf73 | 2.48871283 | 2.588801354 | 10.31191249 | 8.008789156 |
